# Early antiretroviral therapy on reducing HIV transmission in China: strengths, weaknesses and next focus of the program

**DOI:** 10.1038/s41598-018-21791-2

**Published:** 2018-02-21

**Authors:** Pengtao Liu, Zhenzhu Tang, Guanghua Lan, Qiuying Zhu, Huanhuan Chen, Yinghui You, Xiaoyi Yang, Shujia Liang, Yi Chen, Hui Xing, Lingjie Liao, Yi Feng, Zhiyong Shen, Yuhua Ruan, Yiming Shao

**Affiliations:** 1 State Key Laboratory of Infectious Disease Prevention and Control (SKLID), National Center for AIDS/STD Control (NCAIDS) and Prevention, Chinese Center for Disease Control and Prevention (China CDC), Collaborative Innovation Center for Diagnosis and Treatment of Infectious Diseases, Beijing, China; 2 Weifang Medical University, Weifang, Shandong Province China; 3 Guangxi Center for Disease Control and Prevention, Nanning, China; 4 Guangxi Medical University, Nanning, China

## Abstract

Early antiretroviral therapy (ART) initiation is a recommended public health approach for the prevention of HIV-1 transmission. In this cohort study, we included 13132 serodiscordant couples. ART was initiated for patients with CD4+ T cell counts less than 200 cells/uL, 350 cells/uL, and 500 cells/uL respectively. This divided the ART treated couples into three groups. Univariate and multivariate intention-to-treat analyses were performed to examine the association between the study groups. Early-ART initiation was associated with a 45% lower risk of partner infection than was late-ART initiation (AHR 0.55, 95% CI, 0.37–0.81). Mid-ART initiation was associated with a 39% lower risk of partner infection than was late-ART initiation (AHR 0.61, 95% CI, 0.48–0.78). However, the risk reduction between the early and mid-ART groups was not significant. Drug compliance (AHR 1.55, 95% CI 1.03–2.35) and increased baseline viral load (AHR 1.41, 95% CI 1.33–1.51) were associated with an increased risk of infections among partners in the treatment. Prevention of HIV transmission as a result of early ART initiation was feasible on national and regional scales; however, many factors, such as the motivation to commence ART, adherence, and attrition, may affect the impact of this strategy in programmatic settings.

## Introduction

Early antiretroviral therapy (ART) is currently one of the most popular approaches to reduce transmission of HIV-1^1–5^. The findings from the HIV Prevention Trials Network (HPTN) 052 have indicated that early ART can markedly reduce the risk of sexual transmission to HIV-negative sexual partners among heterosexual couples^6,7^. Findings of HIV-1 infection treatment studies also support immediate ART initiation^8,9^. However, the results of the acquired immunodeficiency syndrome (AIDS) and Viral Hepatitis Research (ANRS) 12249 Treatment as Prevention (TasP) study, which was conducted in a rural area of northern KwaZulu-Natal (South Africa), did not show that there was an impact on HIV incidence based on immediate ART initiation^10^. Recently, risk reduction was also reported in an observational study of patients who initiated ART when compared to non-ART in China^1^. The population-level benefits of early ART remain unproven. In order to achieve the World Health Organization’s (WHO) 90-90-90 goal (90% of the people living with HIV know their HIV status, 90% of the people who know their HIV-positive status are accessing treatment, and 90% of the people receiving treatment have suppressed viral loads) for HIV control and prevention, it is worthwhile to assess the factors impacting and limiting the early ART strategy in China.

China’s National Free Antiretroviral Treatment Program (NFATP) began in 2003 and, by the end of 2015, more than 471,140 patients had received ART^11^. Enormous efforts have been made to increase the capability to treat large numbers of people across China. There was an evolution of national ART guidelines following the WHO recommendations^1,12^: when the NFATP was implemented in 2003, the threshold for ART initiation was a CD4+ T cell counts less than 200 cells/uL. In 2008, the NFATP guideline was revised calling for ART initiation among patients with CD4+ T cell counts below 350 cells/uL; this number was further increased to 500 cells/uL in 2010^13,14^. Since 2012, ART has been initiated among all serodiscordant couples regardless of their WHO clinical stage or CD4+ T cell counts. However, to our knowledge, no studies have comprehensively assessed whether the outcomes associated with early ART initiation are feasible and sustainable under programmatic conditions in China.

The Guangxi Zhuang Autonomous Region (Guangxi) is situated in southwest China bordering Vietnam. Guangxi reported 97,389 (cumulative HIV incidence: 0.2%) cases of AIDS and HIV infections until December 31, 2014, representing 15% of the total number of reported HIV and AIDS cases in China^15^. By 2014, heterosexual transmission accounted for 93% of reported HIV infection cases in this region. Research in Guangxi could provide us with reliable information to measure the effect of early ART on the transmission of HIV-1 in China, and thereby assess the feasibility of such an approach in a real-world setting. The aim of this study was to compare the differences in risk reduction in HIV-1 transmission within various populations from programmatic settings, and to analyze the factors affecting the implementation of TasP in China.

## Results

### General characteristics of study participants

Overall, 13132 couples were included in which one partner was HIV-1-positive and the other was HIV-1-negative. Based on the guidelines and WHO recommendations 1339, 7496 and 4297 couples were included in the late, mid, and early-ART initiation groups, respectively.

Demographic characteristics of the participants enrolled in the different ART groups are summarized in Table 1. There were significant differences between the groups based on sex, age, education, marital status, occupation and route of HIV-1 transmission. A greater proportion of other or unknown route of HIV transmission were in late-ART initiation group.

**Table 1:** Baseline characters of the study participants of late, middle and early ART groups.

	Late-ART		Mid-ART		Early-ART		P
	Total No.	Percent (%)	Total No.	Percent (%)	Total No.	Percent (%)	
Total	1339	100%	7496	100%	4297	100%	
Sex							<0.01
Male	924	69.01%	5488	73.21%	3423	79.66%	
Female	415	30.99%	2008	26.79%	874	20.34%	
Age (years)							<0.01
≤30	456	34.06%	1899	25.33%	665	15.48%	
31–40	494	36.89%	2088	27.85%	1129	26.27%	
41–50	257	19.19%	1430	19.08%	1068	24.85%	
>50	132	9.86%	2079	27.73%	1435	33.40%	
Education							<0.01
No schooling	122	9.11%	266	3.55%	122	2.84%	
Primary, secondary school	961	71.77%	6181	82.46%	3629	84.45%	
High school and above	256	19.12%	1049	13.99%	546	12.71%	
Marital status							<0.01
Living with partner	120	8.96%	508	6.78%	181	4.21%	
Married legally	1219	91.04%	6988	93.22%	4116	95.79%	
Occupation							<0.01
Farmer	890	66.47%	5750	76.71%	3601	83.80%	
Other	449	33.53%	1746	23.29%	696	16.20%	
Route of HIV transmission							<0.01
Heterosexual intercourse	925	69.08%	6885	91.85%	4116	95.79%	
Other or unknown	414	30.92%	611	8.15%	181	4.21%	

### Seroconversion rates of late, mid, and early-ART groups

In the late-ART group, 106 HIV transmissions were identified, giving an incidence rate of 8.1 per 100 person-years (95% confidence interval [CI] 6.6–9.6). The rate of transmission in the treated cohort (6.7 per 100 person-years, 95% CI 5.0–8.4) was significantly lower (*P* < 0.05) than that in the treatment-naive cohort (11.2 per 100 person-years, 95% CI 8.0–14.5), with an overall 38% reduction in the risk of HIV transmission in the late-ART group (adjusted hazard ratio [AHR] 0.62, 95% CI 0.42–0.94). In the mid-ART group, 276 HIV transmissions were identified, giving an incidence rate of 4.2 per 100 person-years (95% CI 3.7–4.7). The rate of transmission in the treated cohort (3.1 per 100 person-years, 95% CI 2.5–3.6) was significantly lower (*P* < 0.05) than that in the treatment-naive cohort (5.9 per 100 person-years, 95% CI 5.0–6.9), with an overall 47% reduction in risk of HIV transmission in the mid-ART group (AHR 0.53, 95% CI 0.41–0.68). In the early-ART group, 65 HIV transmissions were identified, giving an incidence rate of 2.7 per 100 person-years (95% CI 1.4–2.4). The rate of transmission in the treated cohort (1.0 per 100 person-years, 95% CI 0.6–1.4) was significantly lower (*P* < 0.05) than that in the treatment-naive cohort (5.3 per 100 person-years, 95% CI 3.6–7.0), with an overall 68% reduction in risk of HIV transmission in the early-ART (AHR 0.32, 95% CI 0.19–0. 54) (Table 2).

**Table 2:** Comparison of seroconversion rates by treatment status.

Group	ART/Non-ART	No. of index patients	Person-years	Serocon-versions	Rate^*^ (95%)	HR(95%CI)	AHR^+^ (95%CI)
Late-ART	Total	1339	1308.99	106	8.1(6.6,9.6)		
	Non-ART^☆^	703	400.05	45	11.2(8.0,14.5)	1.0	1.0
	ART	636	908.93	61	6.7(5.0,8.4)	0.60(0.40, 0.89)	0.62(0.42, 0.94)
Mid-ART	Total	7496	6525.54	276	4.2(3.7,4.7)		
	Non-ART^☆^	4417	2627.26	156	5.9(5.0,6.9)	1.0	1.0
	ART	3079	3898.27	120	3.1(2.5,3.6)	0.51(0.40, 0.65)	0.53(0.41, 0.68)
Early-ART	Total	4297	2430.08	65	2.7(2.0,3.3)		
	Non-ART^☆^	2184	719.74	38	5.3(3.6,7.0)	1.0	1.0
	ART	2113	1710.34	27	1.6(1.0,2.2)	0.30(0.18, 0.50)	0.32(0.19, 0.54)

^*^Rate is number of seroconversions per 100 person-years.

^☆^Non-ART includes pre-treatment groups.

^+^Adjusted for: sex, age, education, marital status, occupation, and route of HIV transmission.

### Hazard ratios for the association between all partner infections and clinical and demographic factors in the study groups (Intention-to-Treat Analysis)

Univariate and multivariate ITT analyses were performed to examine the association between the all partner infections and clinical and demographic characteristic in the study groups. The early-ART group was associated with a 45% lower risk compared to the late-ART group (AHR 0.55, 95% CI 0.37–0.81); the mid-ART group was associated with a 39% lower risk compared to the late-ART group (AHR 0.61, 95% CI 0.48–0.78). However, the risk reduction in early-ART and mid-ART groups was not significant. Additionally, increased baseline CD4+ T cell counts were associated with lower rates of infections among partners (AHR 0.90, 95% CI 0.86–0.94), while an increased baseline viral load was associated with more frequent partner infections (AHR 1.41, 95% CI 1.33–1.51). Drug compliance (missed doses in the last month) was associated with an increased risk of infections among partners in the treated group (AHR 1.55, 95% CI 1.03–2.35) (Table 3).

**Table 3:** Hazard ratios for the association of all partner infections in study groups, clinical factors, and demographic factors.

**Factors**	**HR(95% CI)**	**AHR** ^+^ **(95% CI)**
Early-ART vs. Late-ART	0.58(0.41, 0.81)	0.55(0.37, 0.81)
Mid-ART vs. Late-ART	0.68(0.54, 0.85)	0.61(0.48, 0.78)
Early-ART vs. Mid-ART	0.74(0.56, 0.98)	0.81(0.61, 1.08)
Baseline CD4+ T cell counts (per 100 cells/uL CD4 increment)	1.01(0.97, 1.04)	0.90(0.86, 0.94)
Baseline viral load (per unit log_10_ increment)	1.42(1.33, 1.51)	1.41(1.33, 1.51)
Missed doses in the last month	1.63(1.08, 2.45)	1.55(1.03, 2.35)

^+^Adjusted for: sex, age, education, marital status, occupation, and route of HIV transmission.

### Comparison of seroconversion rates, hazard ratios and attrition rates based on CD4+ T cell counts

In order to determine the relationship between the CD4+ T cell counts and HIV transmission, all serodiscordant couples were divided into four groups according to the CD4+ T cell counts (0–199 cells/uL, 200–349 cells/uL, 350–499 cells/uL, ≥500 cells/uL). We found that the rate of seroconversions increased with decreased CD4+ T cell counts in the non-ART groups. The highest rate of seroconversions was among those in the group with CD4+ TCD4+ T cell counts of 0–199 cells/uL (5.4 per 100 person-years, 95% CI 4.0–6.9), and the lowest rate was among those in the group with CD4+ T cell counts ≥ 500 cells/uL (3.0 per 100 person-years, 95% CI 2.2–3.8). The magnitude of the effect of ART on HIV-1 transmission risk was greatest for couples with CD4+ T cell counts ranging from 200–499 cells/uL (200–349 cells/uL, AHR 0.36, 95% CI 0.24–0.55; 350–499 cells/uL, AHR 0.33, 95% CI 0.17–0.64). However, protection as a result of treatment was not significant in the group with CD4+ T cell counts ≥ 500 cells/uL. The attrition rates were 5.1% (95% CI 4.3–5.9), 9.6% (95% CI 8.0–11.1), 11.6% (95% CI 8.4–14.7), and 14.9% (95% CI 9.0–20.7) in the groups with CD4+ T cell counts < 200 cells/uL, 200–349 cells/uL, 350–499 cells/uL, and ≥500 cells/uL, respectively (Table 4).

**Table 4:** Comparison of seroconversion rates, hazard ratios and attrition rates by CD4+ T cell counts.

**CD4 (cells/uL)**	**ART/Non-ART**	**No**.	**Person- years**	**Serocon-versions**	Rate* (95%CI)	**HR(95%CI)**	**AHR** ^**+**^ **(95%CI)**	**Attritions (%)**
0–199	Total	5024	10565.66	198	1.9(1.6, 2.1)			
	Non-ART	1681	1011.18	55	5.4(4.0, 6.9)	1.0	1.0	
	ART	3343	9554.49	143	1.5(1.3, 1.7)	0.44(0.32, 0.61)	0.47(0.34, 0.65)	160(5.1)
200–349	Total	3370	5200.20	98	1.9(1.5, 2.3)			
	Non-ART	1719	1064.48	51	4.8(3.5, 6.1)	1.0	1.0	
	ART	1651	4135.72	47	1.1(0.8, 1.5)	0.35(0.23, 0.54)	0.36(0.24, 0.55)	147(9.6)
350–499	Total	2257	2543.10	79	3.1(2.4, 3.8)			
	Non-ART	1742	1476.34	68	4.6(3.5, 5.7)	1.0	1.0	
	ART	515	1066.76	11	1.0(0.4, 1.6)	0.36(0.19, 0.68)	0.33(0.17, 0.64)	52(11.6)
≥500	Total	2198	2377.93	61	2.6(1.9, 3.2)			
	Non-ART	1984	1876.30	56	3.0(2.2, 3.8)	1.0	1.0	
	ART	214	501.63	5	1.0(0.1, 1.9)	0.67(0.27, 1.70)	0.62(0.24, 1.58)	25(14.9)

^*^Rate is number of seroconversions per 100 person-years.

^+^Adjusted for: sex, age, education, marital status, occupation, and route of HIV transmission.

## Discussion

In the ITT analysis, we noted a 45% lower risk of HIV-1 infection among partners in the early-ART group than in the late-ART group and a 39% lower risk in the mid-ART than in the late-ART group. Early ART significantly blocked the HIV transmission in discordant couples in a public health intervention program in China, indicating both personal and public health benefits of this treatment model. The HPTN 052 study provided an absolute number to the prevention benefits of early ART among serodiscordant couples – a reduction in transmission of 96%^7^. A lower level of risk reduction was observed in this study. However, the results of this study were better than those from the large scale clinical trial of ANRS 12249 in South African, which showed that immediate ART initiation did not have an effect on HIV incidence^10^. The Chinese government aims to achieve a significant reduction in HIV incidence and HIV-related mortality by adopting the World Health Organization’s 90-90-90 policy. For many years, ART was delayed until a patient’s CD4 count fell below 200 cells/uL in China, which led to the frequent occurrence of opportunistic infections^13,14^. To our knowledge, this is the first large observational study in China assessing the outcomes of early ART over the past ten years. The results of this study could be extrapolated, suggesting that the expansion of the use of early ART could reduce the spread of HIV in China.

It is worth noting that, no significant reduction in transmission risk was observed between the early-ART and mid-ART group in this study. Nonetheless, the study provides numerous lessons learnt about the challenges of achieving treatment as prevention at a population level. In particular, this study demonstrates that the treatment was effective in a CD4 threshold of 200–350 cells/uL, with the impact reducing with increased CD4 threshold of 350–500 cells/uL or greater. Prevention of HIV transmission with early ART was feasible on a national and regional scale; however, the full benefits of this strategy will probably need universal access to very early ART and excellent adherence to treatment. Drug compliance (missed doses in the last month) (AHR 1.55, 95% CI 1.03–2.35) and increased baseline viral load (per unit log_10_ increment) (AHR 1.41, 95% CI 1.33–1.51) were associated with an increased risk of infections among partners in the treatment. In this study, the time of ART treatment was stratified by CD4+ T cell counts, therefore the early, mid, and late treatment groups had significant differences in their CD4+ T cell counts. Lower CD4+ T cell counts account for immuno-deficiency and uncontrolled viral loads, and may also contribute to increased HIV infection in the late group compared to the other groups. After controlling for viral load, the decreased risk in early-ART and mid-ART groups increased, however this was not significant (AHR 0.79, 95% CI, 0.61–1.03, P = 0.09). These findings highlight the challenges of attaining long-term viral suppression in HIV-infected individuals who will be taking life-long ART, so as to reduce risk of infection and prevent spread of AIDS. Rapid expansion of ART without assuring viral suppression can undermine ART effectiveness and contribute to negative public health outcomes, such as the emergence of HIV drug resistance. Development of inexpensive point of care tests for viral load monitoring could permit real-time viral load testing that might allow targeted ART provision to those who have higher CD4+ T cell counts and high viral loads. This might assist in addressing the known relationship between viral load to HIV-1 transmission risk^6,16^.

The rates of HIV transmission through heterosexual intercourse were 69%, 91%, and 95% in the late, mid, and early groups respectively, thus controlling for the route of transmission for 45% and 39% risk reductions was also important. China has come a long way in the provision free ART, coupled with substantially escalated efforts and scaling up of many crucial services in the past ten years^17,18^. In the late-ART group, many patients (31%) were former plasma donors, blood transfusion recipients and injecting drug users (IDUs), as China had the largest number of IDUs in the world in this time^19^. In the mid and early groups, sexual transmission was the major route of transmission. These factors, along with early ART, have all contributed to the risk reductions.

A key element in the serodiscordant couples’ prevention strategy is the use of early ART. However, translation of research findings into public health practice represents an exciting prospect which comes with many challenges. First, not all HIV-infected individuals can be located in time, especially people with acute and early infection who are most contagious in limited healthcare resources^20^. Second, in China, more than half of HIV-1 infected people live in rural areas^12^. HIV-1 infection is difficult to identify and treat immediately if the infection is not in its advanced stage in the vast majority of rural areas^21^. Socially, those most likely to transmit HIV are often among the most stigmatized groups in society^21,22^, and such HIV-infected individuals are not easy to find and treat. In order to get more information, a stratified random sampling technique was used according to the participants’ living area. Most characteristics of the index patients from the two groups were similar, however there were some notable differences. The percentage of regular use of condoms was 37% in seroconversion couples, and 72% in serodiscordant couples (unpublished data). This suggests that we have to conduct qualitative studies to gain an in-depth understanding of reasons behind the intentional practice of unprotected sex among these HIV-infected individuals. Additionally, there is a need for higher scientific priority on finding means to identify infections in rural areas early, given the public health threat they pose on transmission.

The highest attrition rates (14.9%, 95% CI, 9.0–20.7) were found among individuals with CD4 ≥ 500 cells/uL. This finding suggests that education on adherence must be strengthened to control the higher rate of ART attrition when CD4+ T cell counts were above 500 cells/uL. The 2013 WHO guidelines for ART recommended initiation at CD4+ T cell counts 500 cells/uL and immediate ART initiation among specific groups including serodiscordant couples. Several countries have already revised the CD4 threshold to above 500 cells/uL^23^. Recently, another study based on a large ART treatment database in Guangxi showed that HIV infected patients with high CD4+ T cell counts at the time of ART initiation may be at greater risk of treatment attrition^24^. By the end of 2015, a total of 41013 patients terminated ART in China, accounting for 8.7% of the total number of NFATP within China. Attrition, even with the degree observed in our study, can affect further transmission of HIV and HIV resistant drug variants, and lead to premature morbidity and mortality^25^. Therefore, effective strategies to promote retention in HIV care programs are needed when early ART programs are implemented on a large scale in resource-limited settings.

Although HIV transmission from patients with AIDS appears to be most common, the results from this and other studies emphasize that HIV-1 can be transmitted from infected persons who are asymptomatic or minimally symptomatic and who have high CD4+ T cell counts^6,26–28^. Most HIV-1-positive patients CD4+ T cell counts raise the longer they are on ART. If the CD4+ T cell count remains low, the transmission of HIV-1 will continue.

Our study had several limitations. First, the number of seroconversions in the early-ART group was small, and this could lead to insufficient statistical power for the adjusted analyses. Second, the follow-up time on ART in this study was short, relative to the lifetime duration of ART. As such, the long-term protection efficacy of ART needs to be confirmed in additional studies: future studies need statistical power to measure the long-term protection against transmission overtime, including sufficient numbers of seroconversions over the observational period. It is also essential to obtain reliable information about the comparative long-term transmission benefits and behavioral risks associated with ART, particularly ART initiated at higher CD4+ T cell count levels.

Our results strongly indicate feasibility of early ART in the prevention of HIV transmission in serodiscordant couples within programmatic settings. This study also indicated that higher viral loads and poor adherence can enhance the risk for HIV transmission. Our study provides new evidence for the effectiveness of the public health TasP strategy. These findings support the implementation of the WHO’s 90-90-90 strategy in developing countries.

## Methods

### Study design and study population

This retrospective observational cohort study was conducted primarily in Guangxi, and used data collected from January 1, 2003 to December 31, 2014. Individuals were included if they were: aged 18 years or older; sexually active; HIV-negative; married to or cohabiting with a HIV-positive partner; willingness to participate in this study and provide written informed consent.

### Study Definitions

ART was initiated among participants with CD4+ T cell counts less than 200 cells/uL (late-ART group), 350 cells/uL (mid-ART group), and 500 cells/uL (early-ART group) according to the evolution of national guidelines and WHO recommendations. In the late-ART group individuals were also recommended to initiation ART regardless of WHO clinical stage. In the early-ART group, ART was expected to be initiated among all index-partners regardless of WHO clinical stage or CD4+ T cell counts. Attrition was investigated in the 6-month cohort and was defined as the proportion of patients in each group no longer receiving ART at their initiating facility.

### Data Collection and Variables

At baseline and follow-up visits, both HIV-positive and HIV-negative spouses were asked about their demographic characteristics including gender, age, education, marital status, occupation, and the most probable route of infection. The local CDC followed up on these couples every 6 months: HIV-positive individuals were followed up for repeated CD4+ T cell counts and clinical evaluation; HIV-negative partners were tested for HIV seroconversion.

### Statistical analysis

During the follow-up, the treatment effectiveness of ART in HIV-1 acquisition among HIV-negative partners was assessed using the extended Cox regression model with treatment status as a time-varying covariate. Intention-to-treat (ITT) analysis was used in comparing the risk differences between the groups (early-ART versus late-ART, early-ART versus mid-ART, and mid-ART versus late-ART). We calculated the relative hazard of primary outcomes for treatment by subgroups (baseline CD4+ T cell counts, sex, age, and duration of follow-up of the index case). Couples who were lost to follow-up (including couples who separated during the follow-up period) or in which either partner died, were right-censored on the date of last contact. A two-sided *P*-value of 0.05 or less was considered significant. The epidemiology and treatment databases were double-checked in both a relational database designed in SQL Server 2008 and in SAS 9.4. Statistical analyses were conducted using SAS 9.4.

### Ethical approvals

All subjects provided written informed consent to participate in this study. This study and all methods were approved by the institutional review board (IRB) of the National Center for AIDS/STD Control and Prevention, China CDC, and Guangxi Center for Disease Control and Prevention. All methods were performed in accordance with the relevant guidelines and regulations.

### Data availability statements

All data generated or analysed during this study are included in this published article.
